# The Early Stage of COVID-19 Outbreak in Greece: A Review of the National Response and the Socioeconomic Impact

**DOI:** 10.3390/ijerph18010322

**Published:** 2021-01-04

**Authors:** Timokleia Kousi, Lefkothea-Christina Mitsi, Jean Simos

**Affiliations:** 1 Global Studies Institute, University of Geneva, 1205 Geneva, Switzerland; 2 Department of Veterinary Medicine, Aristotle University of Thessaloniki, 54627 Thessaloniki, Greece; leamitsi@gmail.com; 3 Division of Environmental Health and Health Promotion, Faculty of Medicine, Institute of Global Health, University of Geneva, 1205 Geneva, Switzerland; jean.simos@unige.ch

**Keywords:** Greece, Covid-19, SARS-CoV 2, coronavirus outbreak, mathematical modeling, pandemic, public health measurements, social measurements

## Abstract

Greece is a European-Union country, of around 10 million people, located in the southeast part of Europe. The economy is recovering from a long period of deep recession, due to the economic crisis that started in 2008. The economic problems greatly influenced the structure and resources of the healthcare system of the country. In addition to the economic challenges, the country has been facing a refugee crisis, characterized by many overcrowded hotspots and tensions with neighboring Turkey. The COVID-19 outbreak arrived in Greece on 26 February 2020, at the time that Athens had declared a state of emergency at the Greek/Turkish border. From this point in time the government enforced a series of measurements, aiming to contain the epidemic and avoid the collapse of the healthcare system. The vast majority of the general population complied to the measures and consequently Greece’s death toll was low. The impacts of the outbreak are expected to be, as everywhere worldwide, multifaceted and to affect many parts of the economic, social and political life of the country.

## 1. Introduction

The coronavirus disease COVID-19 is an infectious disease caused by coronavirus-2 (SARS-CoV 2). The symptoms vary from mild manifestations of respiratory infection to severe disease. People of old age and those with underlying medical issues are at risk of developing serious disease [1]. The virus initially appeared in December 2019 in the city of Wuhan, China and although scientists have yet to determine its source, it is believed that the virus has a zoonotic origin [1]. On 30 January 2020 the World Health Organisation (WHO) declared the COVID-19 a Public Health Emergency of International Concern (PHEIC) [2] and a pandemic on 11 March 2020 [3]. On 11 April 2020, therapy remains symptomatic and there was is no vaccine available [1].

The virus started spreading progressively from China to the rest of the world. On 24 January 2020 France announced the first positive cases in Europe, three people who had recently traveled to China [4]. Since then more and more European regions have been affected. It seems that Spain is posing the greatest concern, followed by Italy, Germany and France [5].

Greece confirmed the first positive case on 26 February 2020 [5]. The case of Greece exhibits special interest, as the new scenario requires the country to come up against a difficult situation amid other turbulent dilemmas posed to its people and decision-makers. While Greece has not yet resolved the economic and political crisis of 2008, new challenges transpired at the borders [6]. Despite everything, the government acted early, within a few weeks after the first confirmation of positive cases, making crucial decisions, which proved to be fundamental in controlling the virus outbreak [7].

The purpose of this survey is to investigate the course of the pandemic in Greece and the specific challenges that implies to its health care system response; the strategy and implemented measures adopted to contain the outbreak, and their initial effectiveness; and to briefly prospectively assess the impact that this epidemic poses on the social, economic and political status of the country.

## 2. Core Part

### 2.1. Case Presentation

#### 2.1.1. Demographic, Economic, Geographic, Political, and Climatic Characteristics of Greece

Greece is a European Union country, located in southern Europe. The land is covering a surface of 131,957 sq. km, which consists of the mainland and thousands of islands and islets scattered in both the Aegean and Ionian Sea [8]. Like most countries around the Mediterranean Sea, Greece has a Mediterranean climate characterised by mild to cool wet winters and warm to hot dry summers [8]. The total population of the country is estimated around 10,474 million in 2019, while the population density is 81.3 people per sq. km [9]. The number of adults is up to 8703 million and the percentage of the population aged 65 or over was almost 21.14% in 2018 [10] and it is expected to rise in the coming years, while the annual growth rate is −0.16% [9]. Another important factor that affects the demographics is the economic crisis, which started in 2007 to 2008 and brought to the fore an emigration form, known as brain drain, that of highly skilled people. The Greek Central Bank reports that brain drain resulted in human capital outflow of 223,000 young people during 2008 to 2013 [6].

From an economic perspective, Greece is recovering from a period of deep and prolonged recession. In the beginning of 2020, the country had succeeded in correcting the principal macroeconomic and fiscal imbalances, which were responsible for the economic crisis. Greek economic growth is highlighted by the positive performance of several fundamental indicators, such as the economic sentiment indicator and the business confidence indicator, as well as important developments in the financial sector, which aid the boost in bank liquidity. According to the World Bank, the Gross Domestic Product (GDP) in Greece grew slightly in 2017 after a long period of declines starting from 2009, Scheme 1 [11]. The progress of Greece’s economy is also evident by the rating upgrades in the lists of the big credit rating agencies in the last six months [6].

An additional challenge that Greece struggles to address is the refugee crisis, which started in 2015. More than a million refugees entered the country in the period 2015–2016, seeking international protection. As of January 2015, 57,000 of them remained in the country, living in dangerously overcrowded hotspots [12].

The tensions regarding the refugee crisis in Greece re-appeared on 28 February 2020, when the President of Turkey announced that he would open the borders to Greece for the refugees [13]. As a result, thousands of people arrived at the borders with Greece trying to enter the European Union [14]. In response, Greece declared a state of emergency on 3 March 2020, just six days after the confirmation of the first COVID-19 case in its territory [13]. Moreover, the government decided to suspend temporarily the asylum procedures for asylum seekers, a measure which was lifted on 1 April 2020 [15].

The situation de-escalated due to fears for a possible pandemic and the Turkish government decided to close the borders as a measure against the COVID-19 outbreak [16].

In short, the major challenges that Greece had to face in the beginning of 2020 that were threatening its fragile and recovering economy were the exacerbation of the geopolitical tensions in the eastern Mediterranean region, the intensification of the refugee/migrant crisis, and the instabilities of the global economy [6]. The recent threats due to the coronavirus outbreak have now been added to this context [6].

#### 2.1.2. Healthcare System

The Greek population enjoys high life expectancy at birth, estimated at 81, 4 years of age. However, people at the age of 65 are expected to live only 40% of their lives without any form of disability. There are still inequalities in health related to gender and socioeconomic status. The main cause of death remains diseases of the circulatory system, while lung cancer is the leading cause of cancer deaths. Furthermore, 42% of the total number of deaths is attributed to behavioral risks, such as smoking, alcohol consumption, nutritional risks, and low physical activity [17]. Another important metric considered a major risk for serious COVID-19 illness is obesity [18]. In Greece, 17.3% of the total adult population is considered obese based on a European report published in 2016 [19]. Greece was ranked above the European Union average and ranked the country’s population in the 11th position [19].

The economic crisis had an obvious impact on the health of the people. Greece had the second highest ranking on unmet needs for healthcare in the European Union. One in ten households reported that they were unable to access healthcare when they needed to [17]. More specifically, mental health, expressed in suicide rates and number of cases of severe depression, worsened substantially. In addition, infant mortality, an indicator of the quality of healthcare and socioeconomic status, had been increasing and peaked in 2016 with 4.2 deaths per 1000 births, before reducing again in 2017 [17]. Moreover, several studies documented that higher percentages of vulnerable populations like older and unemployed people reported poor health status [20]. Furthermore, the economic crisis appears to influence infectious diseases as well. Since 2010, Greece has experienced a high burden due to several epidemics: increased mortality from influenza; emergence and spread of West Nile virus; re-appearance of malaria; and increased number of HIV infections [20].

The healthcare system was facing important structural challenges related to the financing, organization and delivery of services long before the financial crisis and was ill prepared to handle the problems caused by it. The principal consequences of the economic recession included decreases in public health budgets with declines in the number of the healthcare workforce and their salaries, decreases in pensions, drop in purchase of medical goods, reforms in the pharmaceutical and social insurance sector, merging of healthcare units, rise of access and corruption problems and inadequate primary healthcare services [21].

On the other hand, the financial crisis stimulated change and consequently the healthcare system has undergone a series of structural and efficiency-oriented reforms from 2010 to 2018. In the past, healthcare coverage in Greece was mostly associated with the employment status of the people. The increase in the number of unemployed people, due to the crisis, revealed a problem in health coverage of this population [20]. This issue was addressed in 2016, after major administrative and legislative work. Currently Greece offers universal health coverage, although accessibility to healthcare services remains challenging for vulnerable population groups [22]. On 11 July 2019 [23] the distribution of Healthcare and Social Insurance numbers to the refugee population was revoked. Since then the available healthcare services diminished, as well as the disposal of medications. In November 2019, the government decided to issue temporary numbers for Alien citizens, but this law hasn’t been activated yet. Consequently, the COVID-19 outbreak and the implementation of restrictions in a national level worsened their situation [22].

Another key change was the creation of EOPYY as the sole public insurer in the country, which replaced the previous fragmented system. In addition, we can observe a series of other changes in the pharmaceutical sector, pricing and reimbursement, transforming purchasing and prescribing methods and guidelines. Besides, a comprehensive reform of primary care is currently in motion [17].

So far, Greece does not have mechanisms for adequate planning and optimal allocation of resources [17]. Most healthcare services are strongly concentrated in large cities and the rural areas are missing both adequate facilities and specialist staff. Moreover, we can observe a big imbalance in the distribution of healthcare personnel, from a geographical and skillset point of view [17]. The financial incentives were not sufficient to recruit and maintain an adequate healthcare workforce in remote areas, which exacerbates inequalities in health services. Also, only 1 out of 16 doctors is a general practitioner [17]. As a result, there are no efficient referral and gatekeeping mechanisms in place [20]. Currently, referral to a specialist is not mandatory and people can have access to them without being registered with a general practitioner [17]. This very specialist-focused health system creates inefficiencies and inequalities in healthcare access [17].

In numbers, in 2017, the country had 4.2 beds per 1000 people [17]. Also in 2012 it was estimated that there were 6 critical care beds per 100,000 people available, one of the lowest rates among European Union countries [24]. Currently, there are various models and methods available to estimate the optimal number of hospital beds in regions [25]. On 2 April 2020, the Deputy Minister of Health reported a total of 902 functioning critical care units in the country, from which 247 will be used only for the COVID-19 cases. He added that if needed the number of critical care beds used for the outbreak cases will be increased to 400 [26].

It becomes apparent that the COVID-19 outbreak hit the country when it’s healthcare system is still vulnerable, there is absence of primary care and the health of the people is compromised.

#### 2.1.3. Epidemiological Situation

**Scheme 2:**
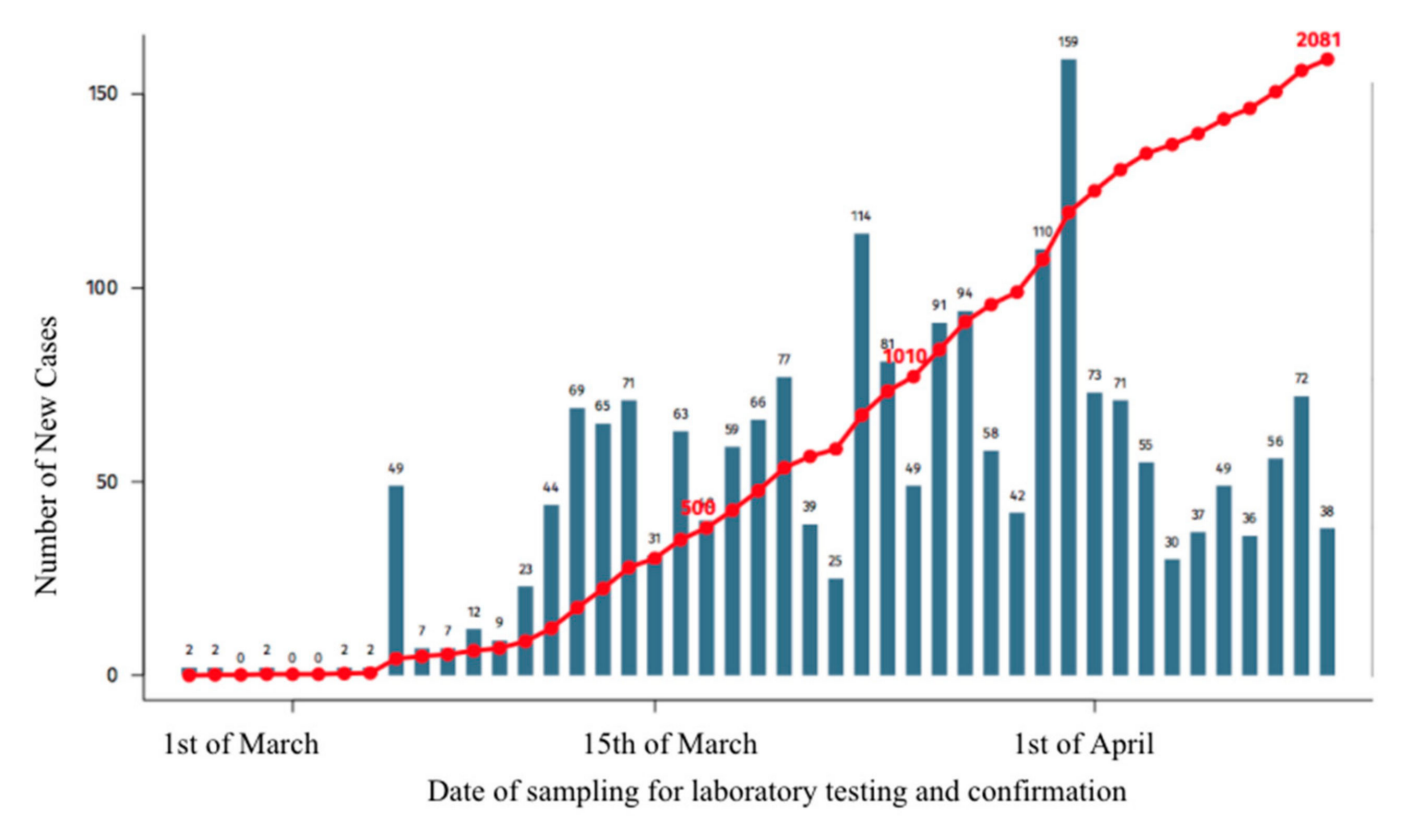
Confirmed COVID-19 cases in Greece as of the 11 April 2020-(the red line shows the cumulative number of confirmed cases) [27].

**Scheme 3:**
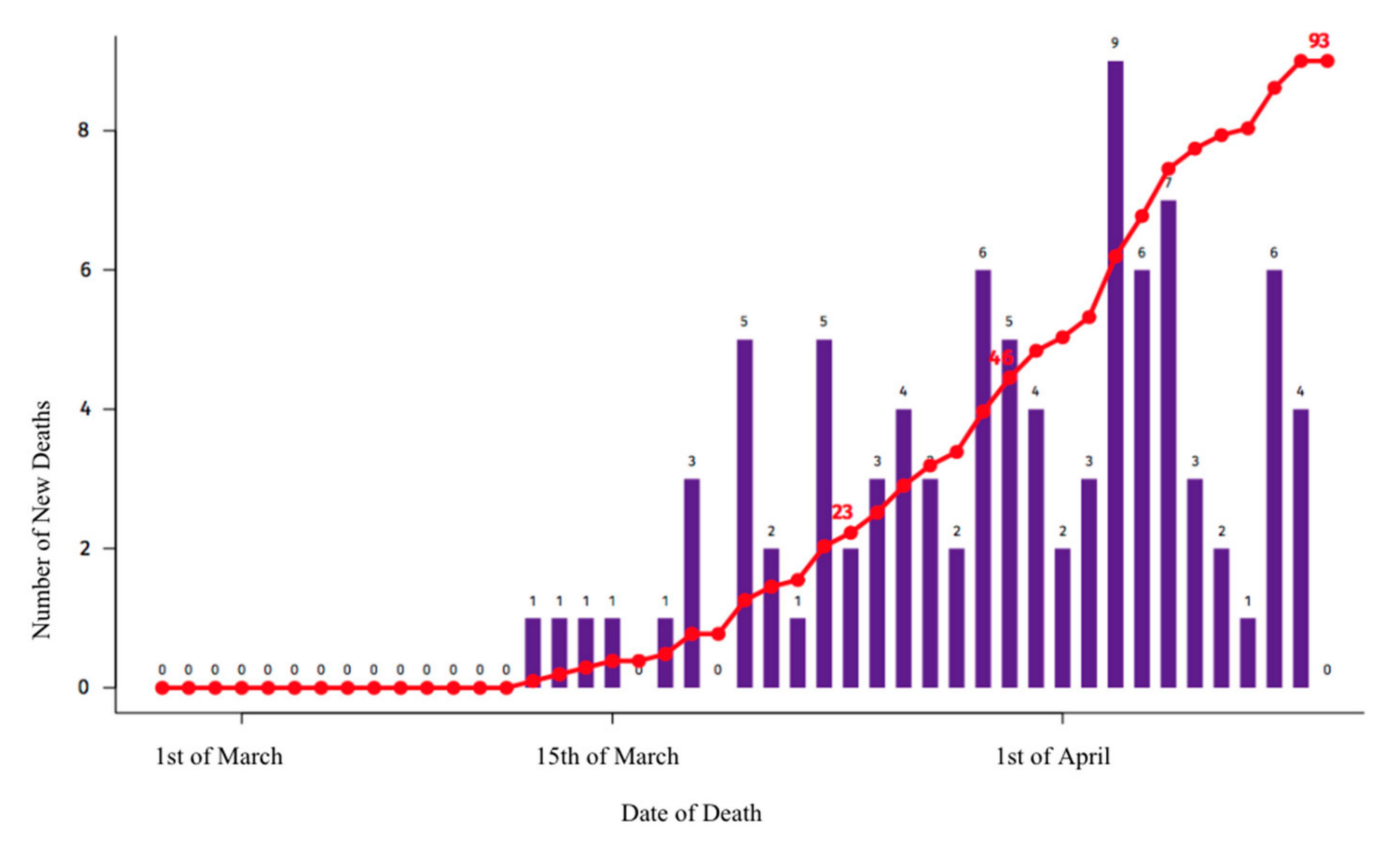
COVID-19 deaths in Greece as of 11 April 2020 (the red line shows the cumulative number of deaths) [27].

On 26 February 2020, Greece reported the first confirmed case of COVID-19 [5] and as of 11 April at 15:00 the total number of confirmed cases has reached 2081, from which 56.8% are men [27], Scheme 2. The daily change of the cases is +3.6%. The total number of deaths since the beginning of the outbreak is 93 and the mean age of the patients that died is 75 years of age, Scheme 3. Greece’s reported numbers represent 198 cases and 8.8 deaths per million people. If we compare with the total number of deaths reported in 2019, then COVID-19 related deaths represent 0.8 deaths per 1000 deaths in 2019. As this article is reporting the epidemiological situation up to April 2020 this number might change a lot till the end of the year [28]. The mean age of the confirmed cases is 49 years of age, while the number of patients that needed intubation is 75 (80% men) [27].

**Scheme 4:**
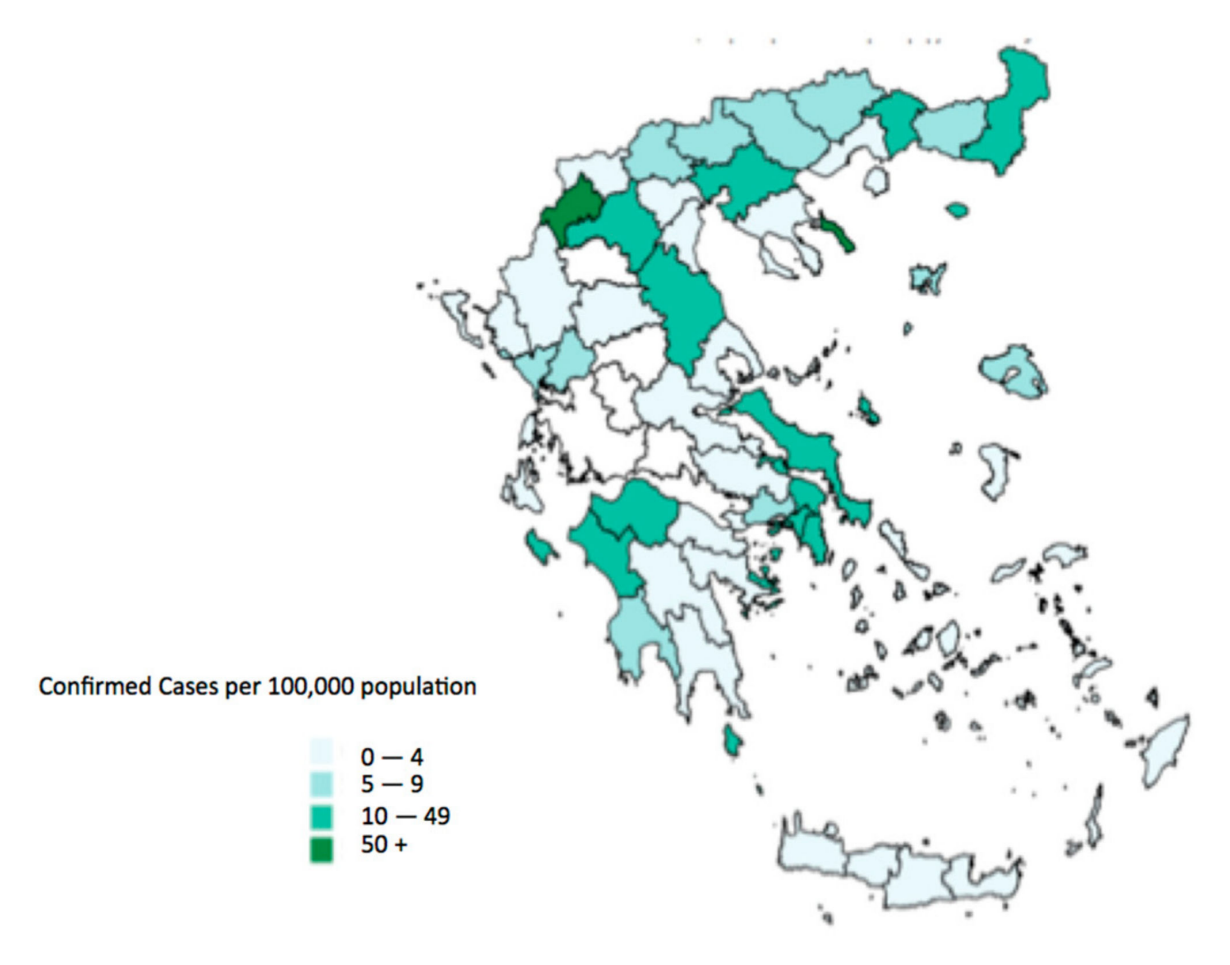
Map of the geographical distribution of confirmed cases as of 11 April 2020 [27].

Out of the 2081 confirmed cases, 535 (25.7%) are connected to international trips, 796 (38.3%) are connected to previously confirmed cases and the rest do not have any known connections or are under investigation [27], Scheme 4. Moreover, there are confirmed cases of refugees living in various refugee camps and hosting facilities that were reported during April [29,30,31].

From 1 January till 11 April 2020, 37,344 samples have been collected and tested for the detection of the SARS-CoV-2 virus, out of which 2417 (6.5%) were positive for the virus [27]. Greece is testing on a scale of 3583 tests per 1 million people [5]. According to the National Organisation of Public Health the criteria for the collection and test of samples for the virus include:

1. Patients with Severe Acute Respiratory Illness that are hospitalised or need hospitalisation,
2. Patients or residents in houses for elderly people that present acute respiratory infection with cough and fever or dyspnea,
3. Healthcare professionals presented with acute respiratory infection and fever,
4. Elderly people (>70 years of age) or people with chronic underlying diseases (such as diabetes, immunosuppressive conditions, chronic cardiac and lung conditions) presented with fever and cough or dyspnea [32].

#### 2.1.4. Management and Outcome

The first confirmed cases of COVID-19 concerned people who had recently returned from virus-stricken regions or their relatives [33]. The government started directly applying protective measures that intensified as the cases escalated.

The first line of implemented measures involved the regions, where the cases appeared. The patients were either placed in quarantine or were hospitalized according to their needs, while their close associates were traced down and examined as possible cases [33]. The Ministry of Health started daily public announcements regarding the contamination progression and issued spots through the media with the precautions the citizens should personally commit to. Mostly the Ministry emphasized the importance of basic self-hygiene rules and the early recognition of symptoms, especially for those that had traveled to affected regions of the world [34]. In addition, the Health Minister revoked all leave for medical staff of the Health Ministry, as well as state-run hospitals across the country until further notice [35].

On 27 February 2020 and within a day after the first cases appeared, the government announced the cancellation of all carnival events [33]. Additionally, in order to prevent mass gatherings limitations applied to the operation of theaters, cinemas, archeological grounds, as well as athletic and art performances [34].

On 8 March 2020, and as the positive cases reached a total of 73, the government banned all school trips nationwide for two weeks [36]. Moreover, elderly centers closed for four weeks, no spectators were allowed to athletic events and conferences were suspended for a four weeks period [36]. It is important to note that the football match between Greek team Olympiakos and English team Wolverhumpton took place also without fans after the government’s decision [37]. In a similar situation a football match in Italy was considered a major reason why the virus spread so rapidly in the city of Bergamo [38]. The aforementioned measures would be put to effect from 9 March and on. It is worth to underline that schools that were temporarily closed would continue the suspension [36].

On 10 Μarch 2020 and while the number of patients reached 89, hospitals restricted the number of visitors, art events with more than 1000 attendants were cancelled and all educational institutes of all levels were closed nationwide for the following fourteen days [39,40]. In an attempt to reinforce the national health system, the Ministry announced that 2000 positions for medical staff would be made available [41].

Moreover, the government issued a two-weeks closure of theatres, courthouses, cinemas, gyms, playgrounds, and clubs starting on 14 March, while on the following day the same restriction applied to malls, department stores, cafes (excluding delivery services), libraries, museums, amusement parks and beauty salons of all kinds [33]. Finally, after long discussions with the Orthodox Church of Greece and despite the persistence of some members of the latter to oppose the national protective actions and ignore the health authorities warnings, all churches agreed to suspend religious services and sacraments from 16 March to 30 March and remained open only for personal prayer and funerals [42].

The refugee camps were considered an important factor in the spreading of the virus and a potential threat to the public, therefore on 17 March the government announced a list of protective actions in order to limit contamination within and outside the camps. Specifically, entrance to the camps was allowed only to employees and all other visitors were banned for fourteen days. Temperature control was mandatory for all new arrivals. Daily announcements and posters in all the spoken languages were utilized for briefing within the camps. All informal educational structures suspended their operation and all other indoor activities were also restricted. Movement in and out of the camp was limited to one person and only for grave causes. Also, special isolation areas were organised in every Reception and Identification center [43].

Lastly, the Region of North Aegean islands which hosts a big number of the refugee camps, decided to create health facilities outside every camp, in order to avoid crowding of the hospitals and maintain access to healthcare amid the outbreak [44].

The International Organisation for Migration emphasized the importance of controlling further spreading of the virus not only within the camps, but also outside, and advised Greek authorities to ensure all refugee camps access to the health care system [29]. On 13 April 2020 and in order to control contamination it was announced that approximately 2000 high-risk people would be transferred from island camps to the mainland, where hotels and other accommodations were organised to receive them [45].

On 18 March 2020, all commercial retailers, apart from pharmacies, supermarkets and gas stations, were to keep their stores closed for at least ten days [33,46]. By that time Greece also banned gatherings of more than ten people and allowed entrance to the country only to EU citizens [27]. At the same time the Ministry of Finance announced a list of supportive measures to aid the national economy [47]. Private businesses that had to suspend their operation would receive an economic aid of 800 euros, while a three-month interest rate subsidy for business loans was also announced [47]. Private businesses also received an amortization postponement until 30 September 2020 [47].

The Deputy Minister of Civil Protection declared that from 22 March 2020 hotels across the country would remain closed until the end of April. This measure excluded three hotels in the cities of Athens and Thessaloniki and one hotel per regional capital [48]. On the same day, the Prime Minister declared to Greek citizens that the whole country was officially under curfew starting from 23 March at 06:00 am until 6 April 2020. Citizens were permitted to move for particular reasons only such as moving to their workplace, helping someone in need, shopping for food or medicine, visiting a doctor or walking a pet. People leaving their home were obliged to carry their ID or passport, as well as a certain certificate stating the reason for moving. Police patrolled the streets and violators were charged a fee of 150 euros [49].

On 28 March 2020 the health authorities announced that patients would receive medical prescriptions via SMS, in order to reduce medical appointments, while the following day the suspension of commercial stores and churches was prolonged until 11 April 2020 [50]. To further support the economy the government declared a 40% reduction in the rent of certain private businesses [51].

On 4 April 2020, the nationwide curfew, issued a few weeks ago, was also prolonged until 27 April. In addition, the government announced the operation of a social support line, offering assistance to those in need, in order to withstand the effects of isolation [52]. As the Orthodox Easter Date approached, the government instructed on 7 April 2020 the citizens to remain home and avoid celebrations. For that reason from 8 April until 27 April 2020, police would supervise highways and other routes and the coast guard would control island connections, so as to limit movement of citizens for Easter celebrations [53]. In that case, the Orthodox Church of Greece supported the government’s decisions and declared that although churches would carry out Easter ceremonies, they would remain closed for the public and urged the people to follow the instructions and stay home [54].

#### 2.1.5. Economical Impact

The economic growth of Greece in 2020 will largely be influenced by the effect of the COVID-19 pandemic on the European and Global economies. There are 3 principal ways that the COVID-19 outbreak is expected to impact the Greek economy, according to the Bank of Greece. From the demand perspective, the outbreak will affect through a deceleration of exports of goods and services (mainly transport, shipping and tourism), as well as the decrease in national consumption and investment [6]. Secondly, from the supply perspective, because of the disturbances in the international and local supply chains of goods, and the closure of businesses, in order to contain the outbreak [6]. It is estimated that small businesses, which employ around 80% of the total workforce have already been facing problems with supplies, liquidity and sales [55]. Finally, on the global financial system side, due to an increase in funding costs, through the reprising of risks, which can lead to stricter financing conditions for banks, business, households and even the Greek State [55].

The growth rate of the Greek economy is expected to decelerate significantly in 2020, as a result of the COVID-19 pandemic. On 11 April 2020, the impact cannot be accurately measured, because of the lack of available data and considering that the pandemic is still in progress [6]. As reported by the bank of Greece, GDP growth is estimated to be zero in 2020, in contrast to 2.4%, which represents the previous estimation [10]. According to the most recent information on the pandemic unfolding, the most probable scenario is that there will be a significant negative effect in the first half of 2020, which will compensate partially in the last half. The slowing down of the Greek economic growth will mainly be a result of the decline in the external and domestic demand of goods and services [6]. The sectors expected to be affected the most are transport, tourism, trade, catering and entertainment [6]. The result of the pandemic in the economy also depends on the fiscal and monetary measures that the government will implement, as well as the measures that will be adopted at an international level. Currently, the European Central Bank has decided to include Greek bonds in the 750 billion euro asset-purchase scheme, whose objective is to aid the economy of the European Union during the pandemic [55].

#### 2.1.6. Social and Political Impact; The Role of Media and Social Media

Early as the first case of COVID-19 appeared in Greece, there was a widespread concern among the people as to what to expect in the near future, which led to an increased demand of everyday essentials such as food, hygiene products and antiseptics [56]. Although the government, as well as the stores, assured that the supplies are efficient and there is no need for trepidation, people queued in the first days outside supermarkets to purchase everyday essentials in large quantities [56]. The COVID-19 frenzy led, early on, to a shortage of masks and antiseptics, while medications containing paracetamol, a substance that seemingly could be used against the symptoms of the virus, also became scarce [57]. Moreover, a few weeks after the first case confirmation, there was a rush of citizens abandoning the cities for more remote regions in an attempt to avoid contamination, something that according to the authorities posed more risks than benefits [58].

On the other hand, despite the overall concern regarding spreading, totally opposite phenomena were also noted. Thus, even though the government and health authorities repeatedly advised against mass gatherings and urged the citizens to stay at home in order to contain the outbreak, many people decided against them and practiced outdoor activities or filled public places like parks and beaches [59].

Another topic that seemed to split the public opinion and gained a lot of attention concerning its management was the operation of churches. Most public buildings had suspended their operation, however despite the government’s instructions, churches continued to practice holy services and worshipers participated in numbers and did not hesitate to receive Holy Communion [60]. Although the authorities warned repeatedly of the risks these practices posed, the Holy Synod continued to preserve their opinion. It was not until 16 March 2020 that the Orthodox Church of Greece agreed to make the churches available only for a limited number of reasons and complied to the health authorities instructions [42]. Resistance of the religious leaders to the implemented public health measures at the early stage of the COVID-19 outbreak has been observed in other countries like Iran, where it resulted in a fierce first wave [61].

The news of COVID-19 appearance in Greece spread quickly as the authorities informed the citizen’s daily of the pandemic through social media. The Health Ministry issued various spots throughout the social media in an effort to alert the Greek citizens to take precautions, but one that standed out the most and became immediately viral was the “Menoume Spiti-campaign” (Staying At Home) [62]. It was initially made public by the Minister of Health through his twitter account and since then has run on every social media platform.

Popular personalities greatly supported the campaign with spots or personal videos through their social platforms. Arkas, a well-known cartoonist urged the people to stay home through his famous characters and even created a new special COVID-19 cartoon series [63]. A group of actors created an online series concerning the necessary isolation the circumstances required [64], while the national television issued an educational series for students to attend to in lieu of school courses [65]. The National Theater also made available online and free of charge performances that were cancelled [66]. Such was the response to the campaign that in April, the government released a new spot that not only urged the people to continue their efforts despite the difficulties, but also praised their patience as well as honored all the people who worked and fought during these hard times [67]. At the European and International level, Greeks are hardly described as law-binding people, but when confronted with the threat of the COVID-19 outbreak, they showed sobriety and discipline [68].

In the refugee camps, the COVID-19 outbreak impaired considerably the daily life of the residents, due to the limitation of mobility inside the camps, the restricted number of visitors allowed and the limited provisions available such as soap and hand-sanitizer. Confine thousands of people in overcrowded facilities, living in unacceptable conditions and having insufficient access to protective equipment, makes it impossible to comply with the regulations and to isolate the confirmed cases [69].

### 2.2. Mathematical Modeling Predictions

**Scheme 5:**
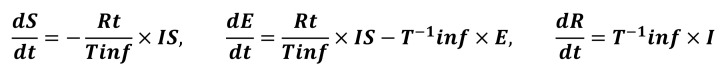
The 4 equations that define the SEIR model.

The researchers used a classical SEIR infectious disease model to simulate the progression of the virus in Greece and compute the maximum number of infections to be expected if the implemented measures fail to contain the transmissions. The presented model was designed based on a simulator created by Gabriel Goh [70]. The SEIR model divides the population of the country into four categories that correspond to the stages of the progression of the disease (Susceptible -> Exposed -> Infected -> Removed). The model is characterised by four equations, which correspond to the stages of the disease progression, Scheme 5. The model subdivides further the Exposed and Infected categories into mild (patients who recover without hospitalisation), moderate (patients that survive after hospitalisation) and fatal (patients that need hospitalisation but do not survive). Each of these variables follows a different path that leads to the final outcome. The model assumes that all fatal cases are admitted to the hospital and all fatalities come from the hospital. Apart from the transmission of the virus, the model enables the computation of the expected death rate and healthcare burden in various scenarios.

In the current simulation, we assume that the population of the country and the rate of infectives remain constant. For simplicity, we assumed a rounded number for the current population of Greece (N = 10,474,000). The initial number of confirmed COVID-19 cases was 2081 and 93 deaths, as of 11 April 2020. For the computations we accept that the length of incubation time (Tinc) is 5.2 days and the duration a patient is infectious (Tinf) is 2.9 days (Kucharski). Also, the Case Fatality Rate is estimated around 4.4 based on total confirmed cases and deaths reported as of 11 April 2020 in Greece. The clinical characteristics were based on the report of the World Health Organisation [71]. More specifically, we assume that the time from end of incubation to death is 32 days and the length of hospitalisation is 28.6 days, while the hospitalisation rate is estimated at 20%. Furthermore, the recovery time for mild cases is 11.1 days and the time to hospitalisation is 5 days.

**Figure 1:**
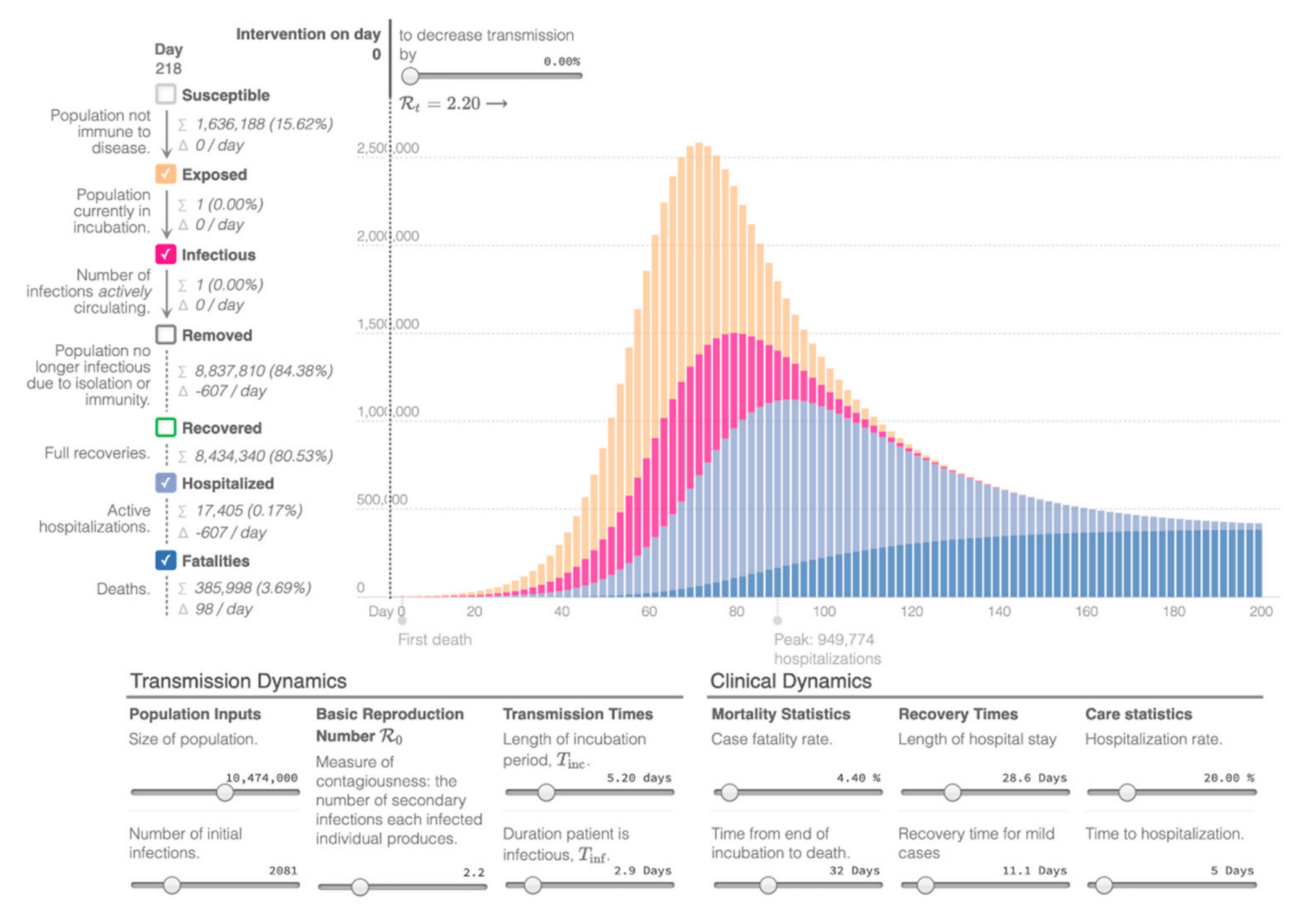
Epidemiological Curve 1.

**Figure 2:**
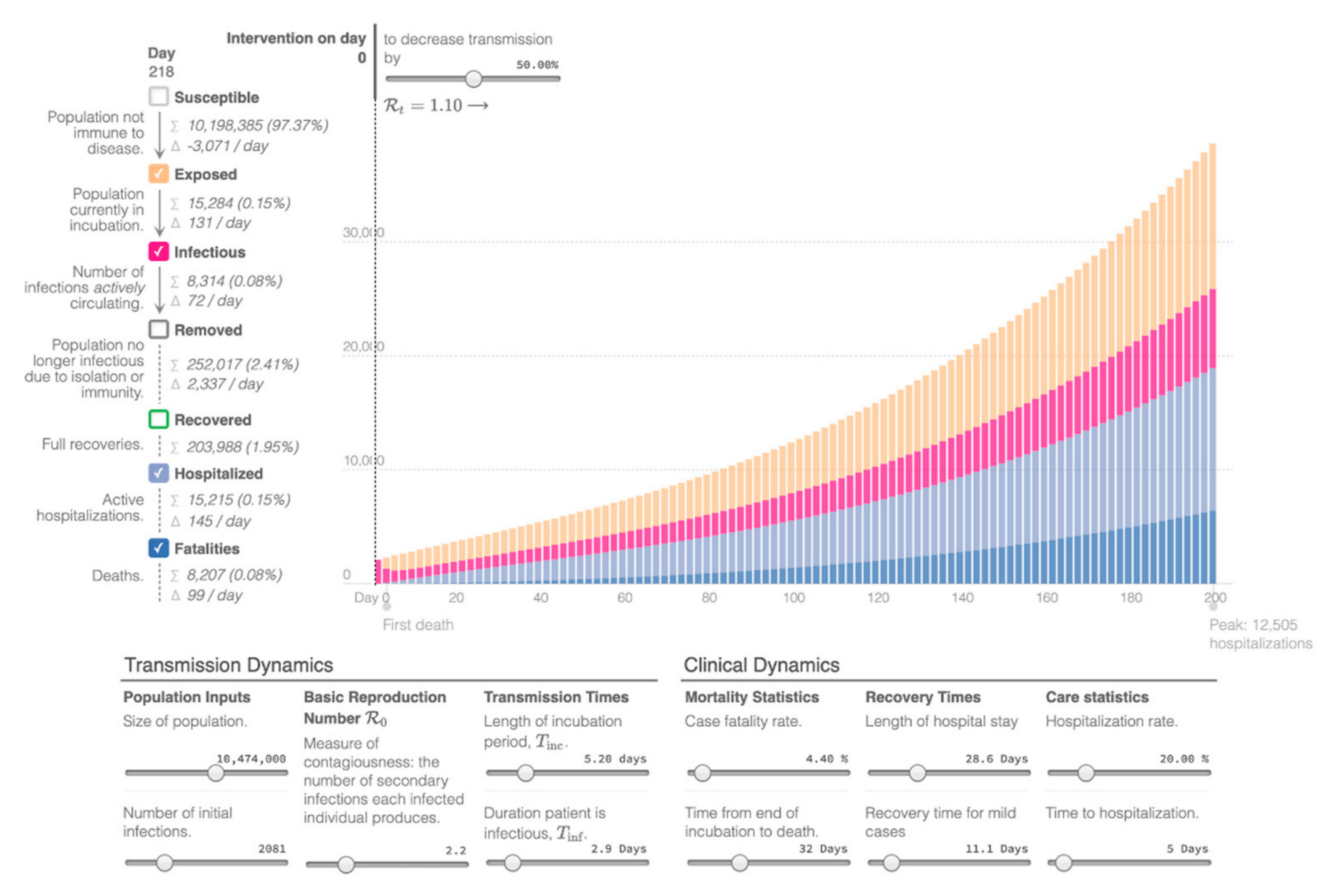
Epidemiological Curve 2.

**Figure 3:**
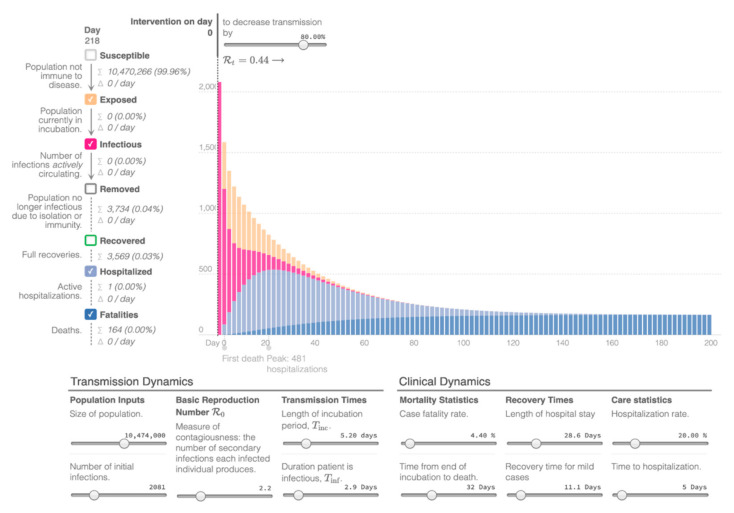
Epidemiological Curve 3.

We present three different scenarios, in which the implemented public health measures have different effects in the spread of the various. In the first scenario, Figure 1, we show the duration of COVID-19 outbreak in Greece if no measures and restrictions were applied. For this scenario we assume that R0 is 2.2 as described in previous studies [72]. In Figure 2, we explore the course of the virus if the implemented measures succeed to decrease the viral transmission by 50% and in Figure 3 we simulate the COVID-19 outbreak, when the viral transmissions decrease by 80%. From the displayed diagrams we have removed the recoveries in order to make it easier to interpret.

The early lockdown that was implemented in Greece allowed to flatten the curve by the end of April, while the daily incident very rarely exceeded 100 cases [5]. Although, if the public health measures failed to control the local transmissions or the general public did not adhere to the restrictions, it is possible that the health authorities would have to face the scenario presented in Figure 1. In this scenario, the healthcare system would have been strained due to the increase in the number of people in need of hospital care (almost a million) by mid of August and the number of deaths could have reached a high peak. Besides, Greece has an estimated total of 628 critical care beds (6 per 100,000 population) available to accommodate the needs of the population. As a result, to avoid overwhelming the healthcare system of the country, the implemented measures needed to maintain a low rate of hospitalization.

In reality, Greece reported 4401 cases by the end of July [5]. It seems that the more plausible projection for the initial course of the outbreak in Greece is depicted in Figure 3, where the R0 is estimated at 0.44 and the restrictions decrease the local transmissions by 80%. We can see that in this scenario the total number of deaths remains low and the number of hospitalised patients do not exceed 500.

In the current published research work we can identify two other publications referring to the same time period that their results are consistent with the predictions we report in the current article. Both of the other publications predict a catastrophic scenario with many deaths in case no public health measures are applied or when the implemented measures decrease substantially [73,74].

The presented mathematical predictions are stressing the need to identify and maintain public health measures that will enable the containment of the viral transmissions in the long term. A prolonged lockdown is not a sustainable solution in the long term, especially for a country whose income is highly connected to tourism. On the other hand, an unstructured lifting of the measures can have grave consequences to the health of the population in the foreseeable future.

## 3. Conclusions and Discussion

In conclusion, Greece’s response to the pandemic has been characterised by efficiency, maturity and early actions, aiming to contain the outbreak. The political system of the country, including Syriza the main opposition party, has responded with control and unity [55]. Although, at the beginning of the outbreak, the government had to focus on the crisis situation in the borders with Turkey and the overcrowded refugee camps in various locations, managed to act in an organised way and flatten the curve early enough [7].

**Scheme 6:**
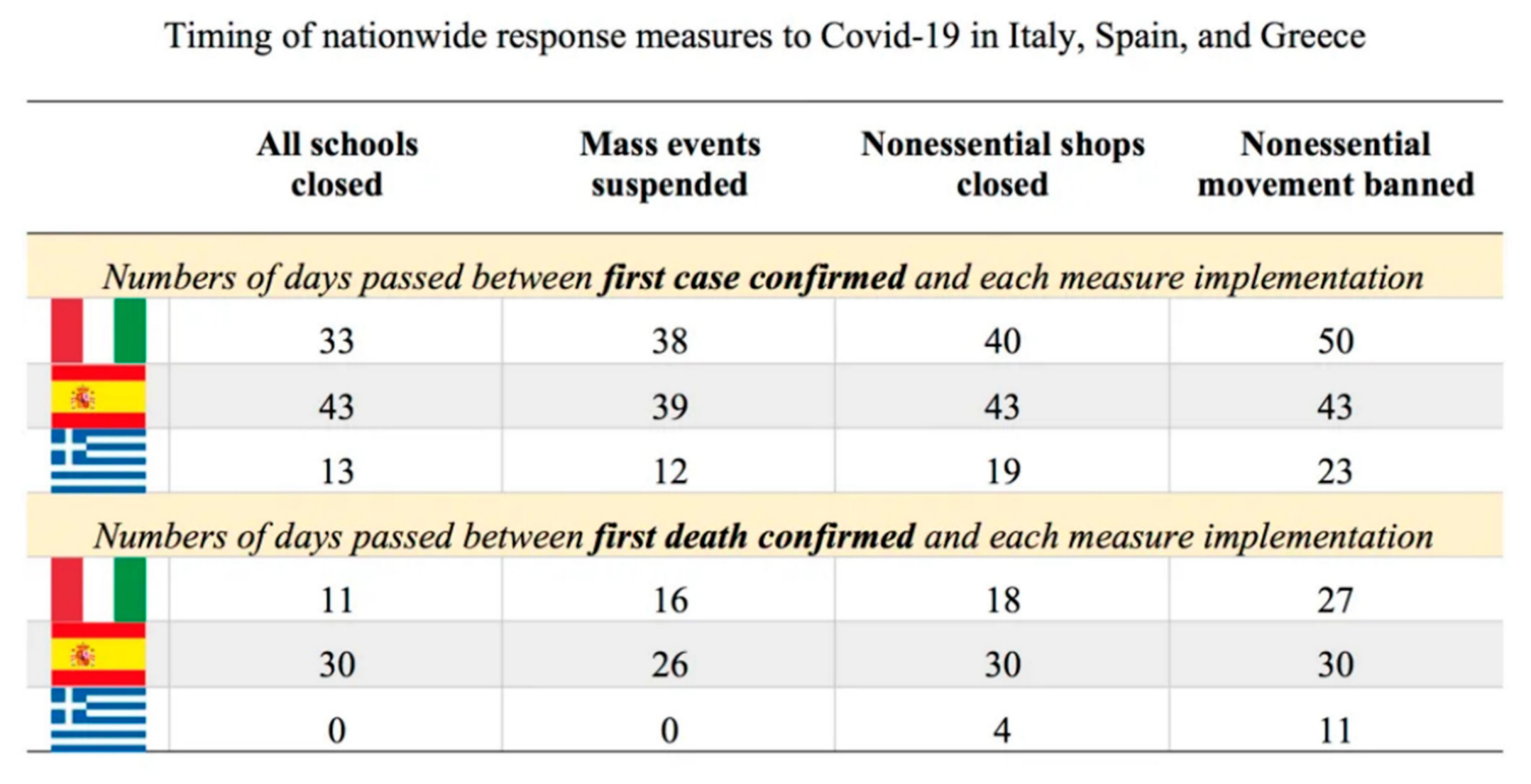
Comparison between Greece, Italy and Spain on the timing of nationwide response measures [7].

In comparison with other European countries, Scheme 6, Greece has implemented measures at an earlier stage of the epidemic [7]. In addition, the general public seemed to be complacent in following the emergency lockdown rules, apart from a few incidents in the beginning of the outbreak [55]. According to the Google Mobility Report, the mobility trends in retail and recreation places (−85%), parks (−70%) and transit stations (−80%) have decreased substantially. There are also drops in the movement around grocery and pharmacy places (−45%), as well as workplaces (−55%) [75].

**Scheme 7:**
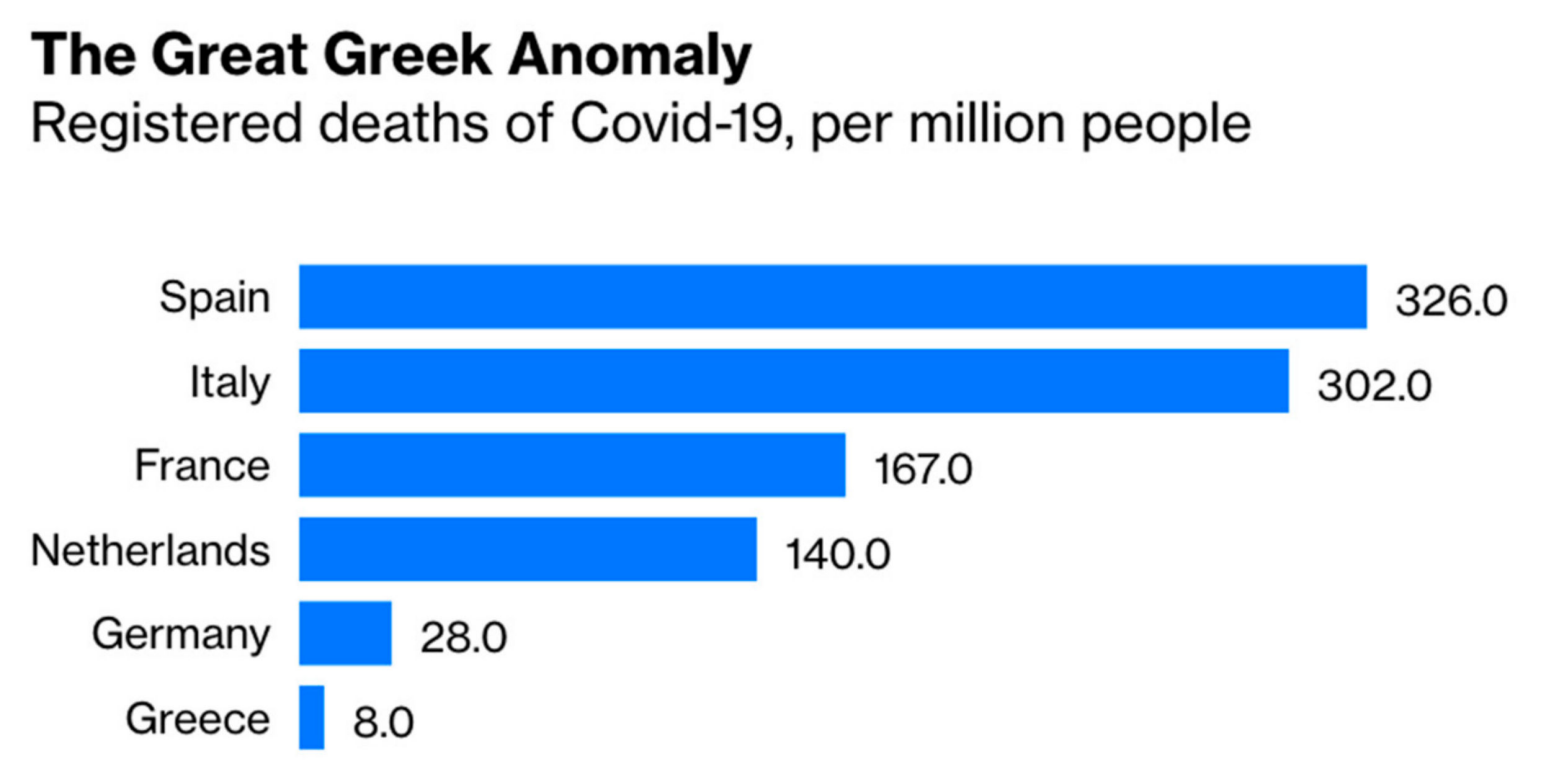
Comparison between Greece and other European countries regarding the registered number of deaths [17].

Consequently, despite the inefficiencies and challenges that the healthcare system is facing, there are no signs that it has been pressured [55]. Also, the total number of deaths per million population remained much lower compared to many other European countries, Scheme 7, at the time of writing [55].

However, the outbreak highlighted a principal limitation, the limited healthcare services available to the refugee population currently living in Greece. Scientists have expressed concerns, regarding the threats that the living conditions in the overcrowded camps pose for the health of the hosted populations. Despite the imposed measures aiming to contain the outbreak, comprehensive strategies need to be designed in order to improve the current state of the refugees. The COVID-19 crisis underlines the immediate need for an action plan, which will provide adequate measures for all. This plan needs to be a collective effort from Greece and the European Union [22].

Rapid decongestion of the overcrowded camps should also be planned. This is a task that needs European coordination and cooperation in order to become successful and ensure descent living conditions for the refugees [22]. Although, in responding to the outbreak, UNHCR suspended all refugee resettlement plans [76] and the European Union changed its political agenda from the refugee crisis to the containment of the outbreak [74]. It becomes apparent that the immediate meeting of the refugees essential needs and the integration of these populations in the European societies needs to become a priority in the Greek and European political agendas [22].

From an economic point of view, the impact of the COVID-19 outbreak could be worrying. As tourism is one of the main industries in the country, prolonged travel restrictions during the summer can hit the economy significantly [55]. On the other hand, the praise that Greece receives on an international level for the country’s response to the outbreak and for protecting the health of the public, is expected to preserve it’s reputation and attract tourists as soon as the measures are lifted. In addition, the government funded the small and medium size businesses that were affected by the pandemic and subsidized the fired workers [75].

Considering everything, it appears that Greece has been a noticeable example of good leadership [55]. The government acted early and upon the advice and plans of scientists and experts; worked efficiently with the other political parties; guided successfully the reaction of the public and did not give in to external pressure from religious and economic interest groups.

## Figures and Tables

**Scheme 1:**
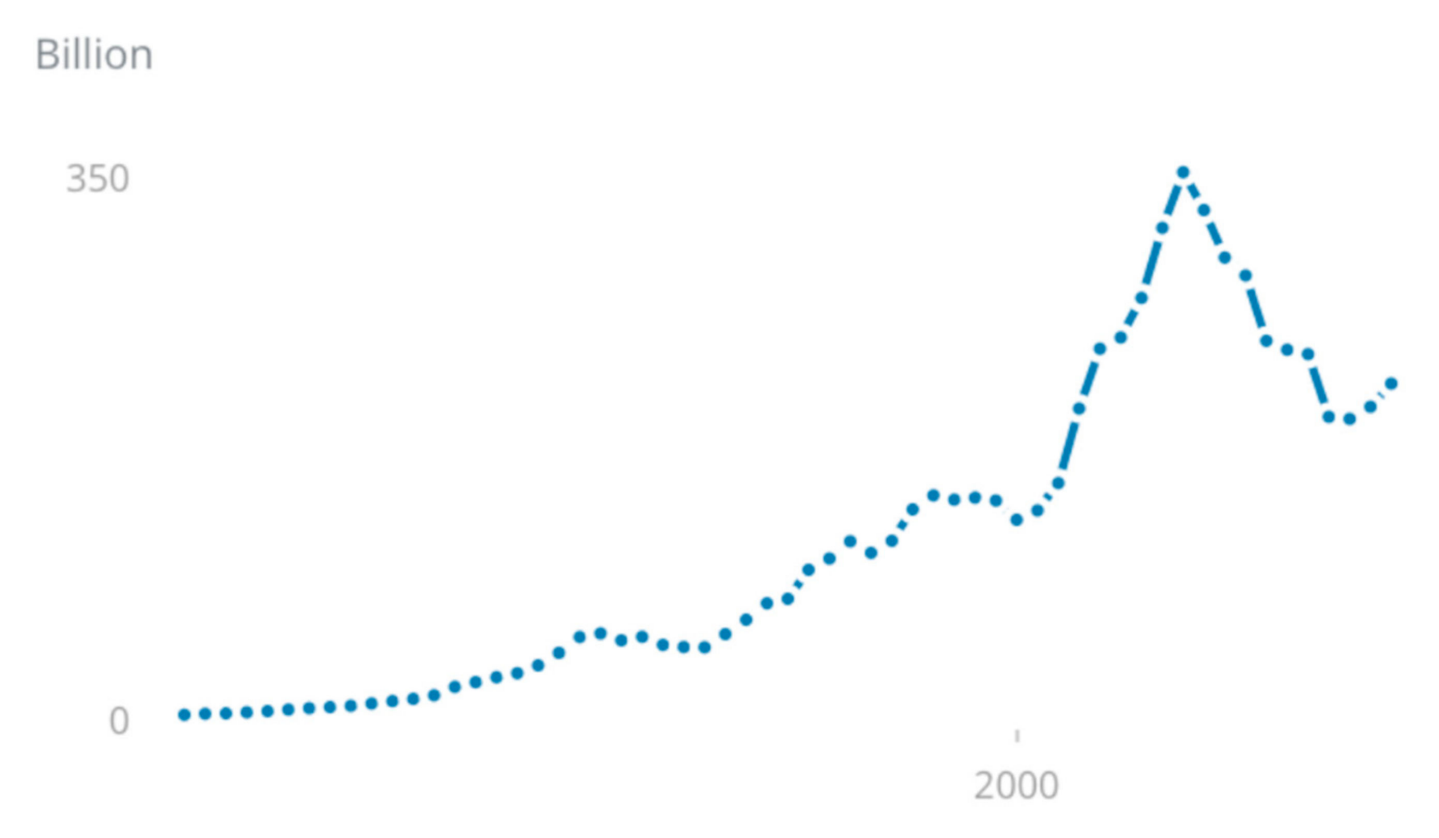
Gross Domestic Product in Greece according to the World Bank [11].

## Data Availability

All data used for the presented research work is publically available.
